# Real-Time Cytotoxicity Assay for Rapid and Sensitive Detection of Ricin from Complex Matrices

**DOI:** 10.1371/journal.pone.0035360

**Published:** 2012-04-19

**Authors:** Diana Pauly, Sylvia Worbs, Sebastian Kirchner, Olena Shatohina, Martin B. Dorner, Brigitte G. Dorner

**Affiliations:** Center for Biological Security - Microbial Toxins, Robert Koch-Institut, Berlin, Germany; Wadsworth Center, New York State Dept. Health, United States of America

## Abstract

**Background:**

In the context of a potential bioterrorist attack sensitive and fast detection of functionally active toxins such as ricin from complex matrices is necessary to be able to start timely countermeasures. One of the functional detection methods currently available for ricin is the endpoint cytotoxicity assay, which suffers from a number of technical deficits.

**Methodology/Findings:**

This work describes a novel online cytotoxicity assay for the detection of active ricin and *Ricinus communis* agglutinin, that is based on a real-time cell electronic sensing system and impedance measurement. Characteristic growth parameters of Vero cells were monitored online and used as standardized viability control. Upon incubation with toxin the cell status and the cytotoxic effect were visualized using a characteristic cell index–time profile. For ricin, tested in concentrations of 0.06 ng/mL or above, a concentration-dependent decrease of cell index correlating with cytotoxicity was recorded between 3.5 h and 60 h. For ricin, sensitive detection was determined after 24 h, with an IC50 of 0.4 ng/mL (for agglutinin, an IC50 of 30 ng/mL was observed). Using functionally blocking antibodies, the specificity for ricin and agglutinin was shown. For detection from complex matrices, ricin was spiked into several food matrices, and an IC50 ranging from 5.6 to 200 ng/mL was observed. Additionally, the assay proved to be useful in detecting active ricin in environmental sample materials, as shown for organic fertilizer containing *R. communis* material.

**Conclusions/Significance:**

The cell-electrode impedance measurement provides a sensitive online detection method for biologically active cytotoxins such as ricin. As the cell status is monitored online, the assay can be standardized more efficiently than previous approaches based on endpoint measurement. More importantly, the real-time cytotoxicity assay provides a fast and easy tool to detect active ricin in complex sample matrices.

## Introduction

Ricin, one of the most poisonous toxins known, is a glycoprotein derived from the seeds of the castor plant *Ricinus communis*. This cytotoxin is highly toxic both to humans and animals [1], [2]. The plant is cultivated as an ornamental and industrial flower all over the world. As by-product during castor oil production, ricin is mass-produced above 1 million tons per year [3]. On the basis of its availability, toxicity, ease of preparation and the current lack of medical countermeasures, ricin has gained attention as potential biological warfare agent and is listed as category B agent of potential bioterrorism risk by the Centers for Disease Control and Prevention (CDC, Atlanta, GA, USA; [4], [5]).

Functionally active ricin consists of two ∼32-kDa subunits, the A-chain and the B-chain, which are linked by a disulfide bond. Both chains are needed for toxic action *in vivo*. The B-chain acts as a lectin, which binds to terminal galactose residues on the eukaryotic cell surface and mediates ricin cell entry by endocytosis [6]. The A-chain is an RNA-specific *N*-glycosidase that hydrolytically removes a specific adenine from the 28S ribosomal subunit, thereby inhibiting the protein biosynthesis and ultimately leading to cell death [7]. As well as ricin, castor seeds also contain a second lectin, *Ricinus communis* agglutinin (abbreviated in the text as agglutinin), which is highly homologous to ricin, but less toxic [8]. Agglutinin consists of a dimer of two associated ricin-like molecules, each of which contains A- and B-chains. The homology at the amino acid level between agglutinin and ricin is around 93% for the A-chains and around 84% for the B-chains [9].

The mortality in ricin poisoning is dependent on the route of administration. In mice, the median lethal doses (LD50) for injection, inhalation or ingestion are reported as 2–10 µg ricin/kg body weight, 3–5 µg/kg or 20 000–30 000 µg/kg, respectively. In humans, the oral LD50 of 1 000–20 000 µg ricin/kg body weight is estimated from accidental ingestion of castor beans [10], [11]. For agglutinin, it is approximated from animal studies that the toxicity is about two orders of magnitude less than that for ricin [12], [13].

Similar to other toxins, ricin acts in the absence of the producing plant and its genetic information. Therefore, it is necessary to detect the protein itself, not only the plant's nucleic acid. Currently, the analysis of ricin is mainly based on immunological methods [14], [15], mass spectrometry analysis [16], [17], or functional *in vitro* and *in vivo* assays (for an overview of the latter see Table S1). In the case of an intentional release of ricin into the environment, the discrimination of functionally active and denatured ricin is important, especially with regard to emergency operating schedules, forensic analysis and therapy. This information can only be obtained from functional assays, which can be principally differentiated into assays detecting the A-chain activity, the B-chain activity, or both. *In vitro* assays, like adenine-release assays or cell-free translation assays based on rabbit reticulocyte lysate, analyze the enzymatic activity of the A-subunit [18], [19], [20], [21], [22], [23], [24]. Glycan binding of the ricin B-subunit is detected by enzyme-linked lectin assays [25]. However, the detection of the activity of the isolated subchains provides no information on the activity of the intact 64 kDa ricin molecule. Therefore, the detection of active ricin requires *in vitro* or *in vivo* assays for both subchains. *In vivo* assays have the advantage that whole-organism responses can be monitored but, on the basis of different species and strain susceptibilities that have been reported for ricin, animal bioassays seem to be difficult to standardize and raise ethical concerns [26], [27], [28]. An alternative are *in vitro* immunocapture assays combined with adenine release measurement [17], [29], or cell-based functional assays. Current cell assays use different endpoint read-outs of cell death via biochemical, fluorescent or radioactive detection [30], [31], [32], [33]. The detection limits for ricin analysis in cell-based bioassays have been described as being between 0.01 ng/mL and 0.8 ng/mL from complex matrices. Cytotoxicity is detected at the end of the assay after different cell cultivation times (ranging from 4 to 28 h), in order to obtain a “snapshot" of ricin action. Taking into account the duration of the assay required to reach high sensitivity, time-dependent toxicity values might be more informative than endpoint measurements. Another drawback of the current cell-based assays is represented by a lack of online and internal viability control. It is only known at the end of the assay if the cells adhere, grow and die, according to a standardized procedure. Therefore cell-based assays usually show a higher degree of variability than other methods. In terms of reproducibility of cell-based assays, it is important to standardize all growth parameters and to include specificity controls (i.e. functionally blocking antibodies) in order to avoid experimental artefacts.

In the present study, we describe an online functional ricin cytotoxicity assay based on a real-time cell electronic sensing (RT-CES) system. The cell proliferation and toxin-induced cell death of African green monkey (Vero) cells is monitored online in the RT-CES system. This system uses an impedance sensor technology to noninvasively and label-free quantify cell viability, based on cell number, morphology and adhesion in real-time [34]. The cells are seeded in E-plates into which microelectrodes are integrated. Low-voltage application leads to the generation of an electric field, which is differentially modulated by the cells. The higher the number of cells attached to the plate surface, the higher the impedance monitored by the RT-CES system as a read-out for cell viability.

The aim of this report was to detect and quantify biologically active ricin in a real-time cytotoxicity assay. The assay duration, sensitivity and specificity was tested for ricin and agglutinin, as compared to other types of lectins. This method allowed for the detection of low concentrations of active ricin in different food matrices and in organic fertilizer, without significant interfering matrix effects. The real-time impedance measurement of adhering, proliferating and, ultimately, dying cells turned out to be highly reproducible, thus opening the door to standardized cell-based cytotoxicity assays.

## Results

### Real-time monitoring of Vero cell growth pattern

**Figure 1:**
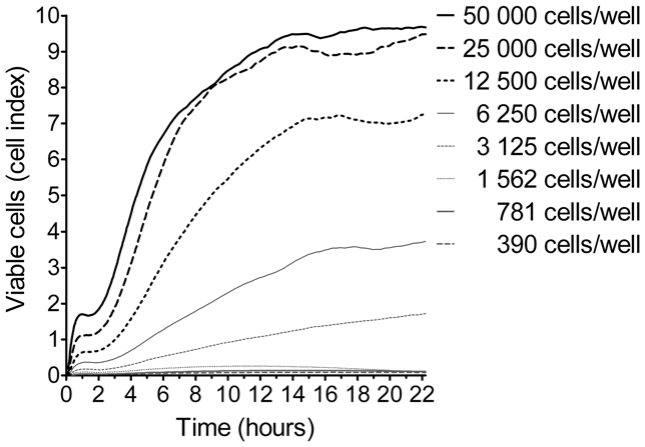
Dynamic monitoring of Vero cell proliferation. Serial dilutions of Vero cells were seeded at indicated densities of 50 000 to 390 cells/well in a 96-well E-plate. The attachment phase, lag-phase and proliferation phase were dynamically monitored every 15 min for 22 h, as indicated in the text. Data shown are representative of three independent experiments showing similar results.

In order to set up a standardized cell-based cytotoxicity assay, the culture conditions and assay procedures were thoroughly optimized. First, the Vero cell growth pattern was dynamically monitored using the RT-CES system, which detects cellular impedance as measure of cell number, morphology and adherence [34]. To this end, we seeded different Vero cell concentrations, from 390 to 50 000 cells/well, into an E-plate and monitored the cell proliferation online for up to 60 h. Cell growth was recorded as cell index (CI), which corresponds to the electrical impedance of a well measured by the RT-CES system [34]. Depending on the number of cells seeded within an E-well, the CI of proliferating Vero cells ranged from 0.5 to 10 (Figure 1). Cells seeded in concentrations below 3 125 cells/well did not proliferate in the observation period shown. Vero cells seeded with 12 500 cells/well or above showed vigorous growth in the first 12 h post-seeding (Figure 1) and reached a plateau after 42 h hours, up to a CI of 10 (data not shown). Characteristic cell proliferation parameters were observed for the optimal cell concentration of 12 500 cells/well, which was chosen for further experiments: The attachment phase of the cells to the plate was completed after 1 h (CI increased from 0 to 0.5), the lag-phase lasted from 1 to 2 h (CI remained at 0.5), the start of proliferation was recorded after 2 h (CI increased over 5), and after 14 h the proliferation of the cells decelerated into the confluent phase (CI remained at 7; Figure 1).

### Measurement of ricin toxicity in the real-time cell system

**Figure 2:**
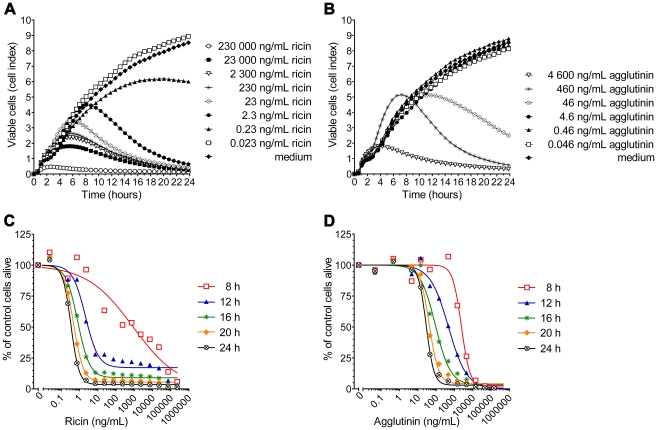
Real-time measurement of cytotoxicity and time-dependent standard curves for ricin and agglutinin. Vero cells were seeded in a 96-well E-plate (12 500 cells/well). Immediately after seeding, cells were exposed to the indicated concentrations of ricin (A, C), agglutinin (B, D), or medium (control). Cell proliferation was dynamically monitored every 15 min for 24 h. Figures A and B show the time-dependent alteration of the CI for different ricin or agglutinin concentrations. Figure C and D display the percentage of viable cells plotted against toxin concentrations at selected time points (conversion of the data from figure [A, B] to [C, D] is described in material and methods). Data shown are representative of five (A, C) or three (B, D) independent experiments with similar results.

We used the optimal cell concentration of 12 500 cells/well to analyze the toxic effect of serial dilutions of ricin (230 000 to 0.023 ng/mL) or agglutinin (4 600 to 0.046 ng/mL) in cell culture medium, respectively. Immediately after the seeding of cells into E-plates, ricin or agglutinin was added to the cells (without prior attachment of the cells to the plate). Electrical impedance of cells within an E-well was monitored using the RT-CES system over 24 h, where attachment and proliferation were visualized as a rise in CI (correlating with rise in impedance) and detachment and cell death as a drop in CI (correlating with drop in impedance). As shown in Figures 2A and 2B, all cells showed a characteristic attachment and lag phase of cell growth, independent of toxin concentration. Notably, the duration of the proliferation phase was dependent on the toxin concentration. The growth curves of Vero cells treated with a toxin concentration of 0.23 ng/mL ricin or 46 ng/mL agglutinin (or higher), respectively, did not reach confluence compared to untreated cells (Figures 2A, 2B). Time-to-inhibition of cell proliferation was dependent on the toxin dose, with the earliest inhibition visible after 3 to 4 hours, at the highest concentrations tested. For ricin tested in concentrations of 2.3 ng/mL or above (or 460 ng/mL agglutinin), a concentration-dependent decrease of CI was recorded after 3.5 to 10 h, reaching a CI below 2 after 24 h. Toxin concentrations of 0.23 ng/mL ricin or 46 ng/mL agglutinin also showed an inhibition of cell growth, albeit after more than 13 h of incubation time (Figures 2A, 2B). Based on these data, Figures 2C and D show dose–response curves at selected time points of the real-time cytotoxicity assay. As expected, the toxic effect of ricin and agglutinin on Vero cells increased with toxin concentration and incubation time. *In vitro* cytotoxicity, where 50% of the cells were alive (IC50), was detected at the earliest after 8 h for 880 ng/mL ricin (Table 1). For agglutinin, the IC50 value at 8 h was higher (2 400 ng/mL). For ricin, sensitive detection was determined after 24 h, with an IC50 of 0.4 ng/mL. At the same time point, agglutinin showed a toxicity that was 75 times lower than ricin (IC50 of 30 ng/mL; Table 1). Upon prolonged incubation for 42 h, the most sensitive detection of ricin was reached with an IC50 of 0.1 ng/mL (and 6.7 ng/mL for agglutinin). Longer incubation times did not significantly result in higher sensitivities (Table 1).

**Table 1:** IC50 values for ricin and agglutinin at different time points of the real-time cytotoxicity assay.

time [h]	IC50 ricin [ng/mL]	IC50 agglutinin [ng/mL]
8	880	2 400
12	3.0	470
16	1.0	100
20	0.5	44
24	0.4	30
30	0.36	26
36	0.17	11
42	0.10	6.7
48	0.08	4.8
54	0.06	4.2
60	0.06	4.1

Since the real-time cytotoxicity assay allows for monitoring the cell proliferation in real-time, the assay can be better standardized than conventional cell-based cytotoxicity tests: even slight changes in cell culture conditions can be visualized and used to optimize the test, as shown in Figure S1: parameters like the cell density prior to seeding cells into E-plates (Fig. S1 A) or the method used to detach cells from the culture flask (Fig. S1 B) quite strongly influence the growth characteristics of the cells, similar to seeding of different cell numbers (Fig. 1).

The precision of the optimized real-time cytotoxicity assay was evaluated by the measurement of cytotoxicity of serial dilutions of ricin after 24 h or 42 h, respectively: for within-run precision serial dilutions of ricin (1 ng/mL to 0.03 ng/mL) were performed in four replicates and measured on one day yielding within-run CVs between 13% and 2% after 24 h incubation (Table S2). For between-run precision serial dilutions of ricin were performed on four different days and the results were used for calculation of CVs. At the 24 h time point, the between-run CVs were determined between 30% and 6% (Table S2).

### Comparison of RT-CES and MTT cytotoxicity assay

**Figure 3:**
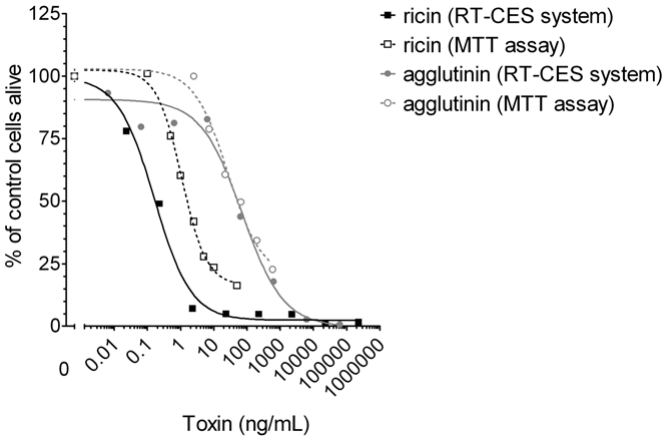
Comparison of ricin and agglutinin cytotoxicity in RT-CES system and MTT assay. Vero cells (12 500 cell/well RT-CES system, 10 000 cells/well MTT assay) were seeded in a 96-well E-plate (RT-CES system) or 96-well cell culture plate (MTT assay), respectively. In the RT-CES system (filled symbols), serial dilutions of ricin (grey) and agglutinin (black) were incubated immediately after cell seeding, and cell proliferation was monitored online for 24 h. For the MTT assay (open symbols), cells were cultivated for 18 h and incubated afterwards with ricin or agglutinin. After 2 h cells were washed and cultured for a further 20 h, before the MTT reagent was used to determine cell viability. Data shown are representative of two independent experiments showing similar results.

In order to compare the performance of the online cytotoxicity assay using the RT-CES system with the classical endpoint cell viability test, we performed parallel assays using ricin or agglutinin. Serial dilutions of ricin or agglutinin were incubated with Vero cells, and cell growth was determined either in real-time format over 24 h (RT-CES system) or as endpoint measurement after 20 h proliferation using an MTT assay. Both techniques resulted in sigmoidal dose-dependent response curves (Figure 3). The IC50 for ricin in the RT-CES system and the MTT assay were similar (0.4 ng/mL and 1.5 ng/mL, respectively). For agglutinin, the IC50 values were in the same concentration range using both assay systems (30 ng/ml for RT-CES system, 50 ng/mL for MTT assay). The duration of the full assay, starting from cell seeding to sensitive read-out, was 24 h for the RT-CES system and 45 h for the MTT assay. On a quantitative level, the RT-CES system reached a similar sensitivity as the MTT assay, but was significantly faster. At the same time the overall precision of the RT-CES system was similar to the precision of the MTT assay (Table S3).

### Specificity of the real-time cytotoxicity assay

**Figure 4:**
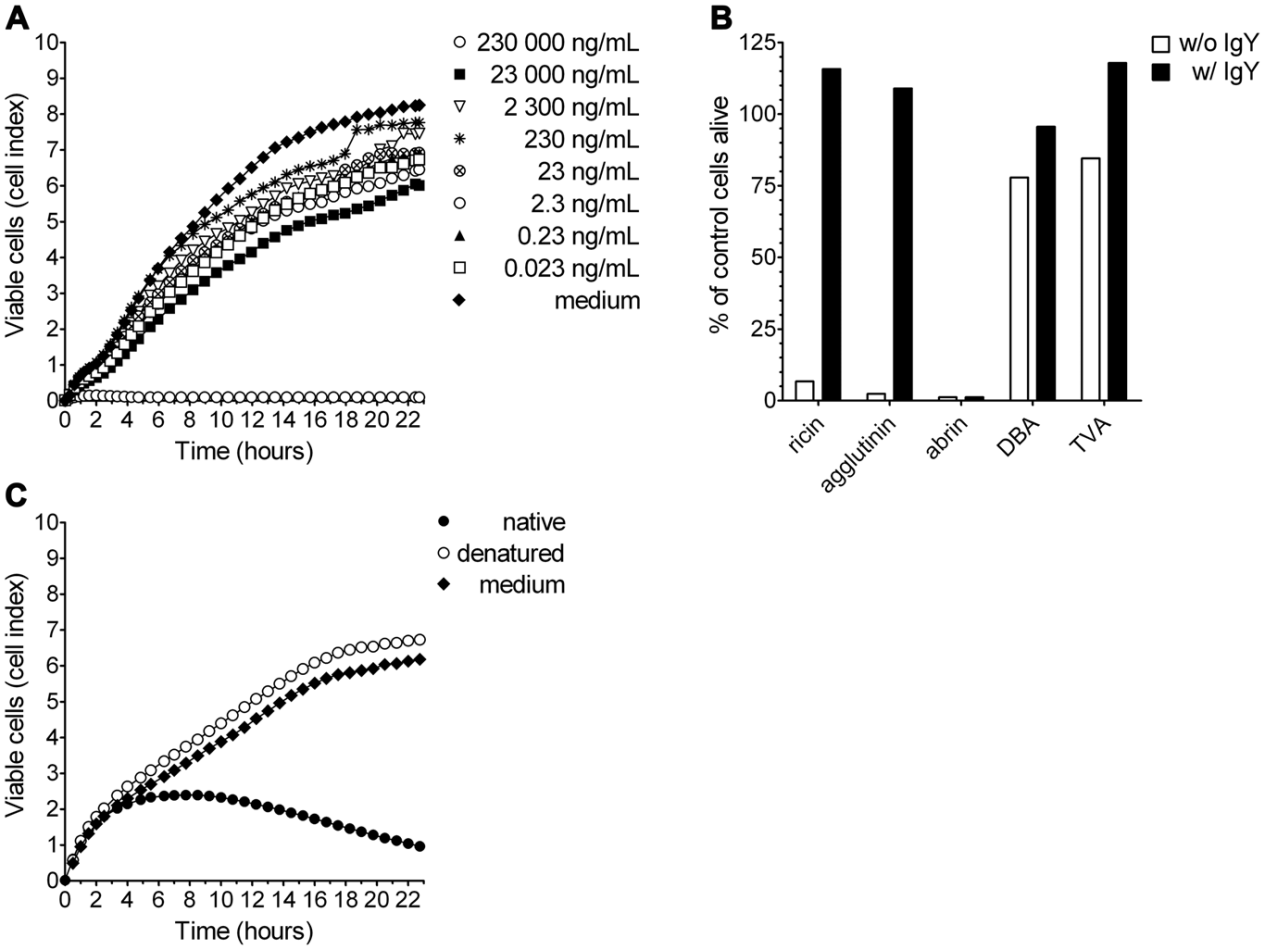
Specificity of the cytotoxicity test. Vero cells (12 500 cells/well) were seeded in a 96-well E-plate. (A) Serial dilutions of ricin were preincubated with polyclonal anti-ricin IgY for 1.5 h at 37°C, and then added to the cells. Cell proliferation was dynamically monitored every 15 min for 23 h. Data shown are representative of five independent experiments showing similar results. (B) Vero cells were exposed to 10 µg/mL ricin, agglutinin, abrin, DBA or TVA, respectively (white columns). In order to show the specificity of the assay, the different lectins were preincubated with anti-ricin IgY as above (black columns). The viability of the cells after 21 h is depicted as percentage of the viability of untreated control cells (100%). Data shown are exemplary data out of two independent experiments showing similar results. (C) Ricin (20 000 ng/mL) was heated in PBS for 30 min at 95°C (denatured, white circles), or was left untreated (native, black circles) and then added to the cells. Cell proliferation was dynamically monitored every 15 min for 23 h. In parallel, cell growth was monitored in medium only (negative control, black diamond). Data shown are representative of two independent experiments showing similar results.

In order to demonstrate the specificity of the assay, ricin (in concentrations ranging from 230 000 to 0.023 ng/mL) was preincubated with chicken anti-ricin polyclonal antibodies (IgY). The action of the toxin on cells was then monitored online for 23 h. Undisturbed cell proliferation, equivalent to a complete block of ricin's functional activity, was observed up to a ricin concentration of 23 000 ng/mL (Figure 4A). For the highest ricin concentration tested (230 000 ng/mL), the IgY concentration was not sufficient to block ricin activity, resulting in cell death.

We further analyzed the specificity of the real-time cytotoxicity assay by comparing the cytotoxic effects of ricin and agglutinin with other plant lectins (*Abrus precatorius* abrin [abrin], *Dolichos biflorus* agglutinin [DBA] and *Triticum vulgaris* agglutinin [TVA]) in the absence or presence of anti-ricin IgY (incubation period of 21 h). Ricin, agglutinin and abrin caused cell death, whereas the plant lectins DBA and TVA showed no significant toxic effects (Figure 4B). As expected, anti-ricin IgY were able to specifically block the functional activity of ricin and agglutinin, but not the activity of abrin. Only functionally active ricin induced cell death, since heat-inactivated ricin had no effect on cell proliferation (Figure 4C).

### Detection of ricin activity in complex matrices

**Figure 5:**
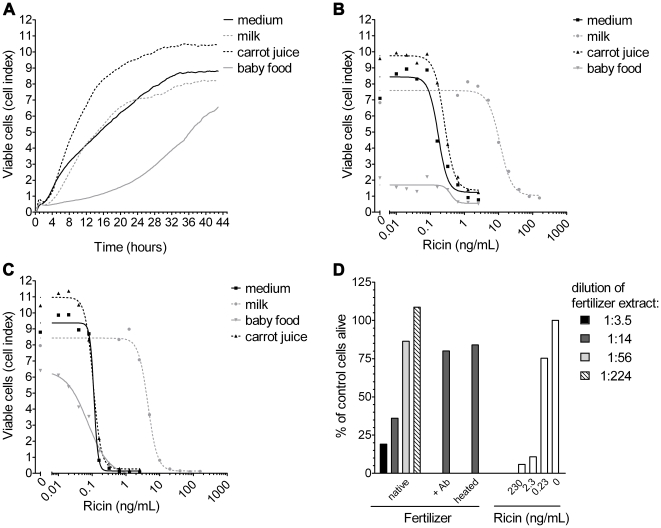
Detection of functionally active ricin in complex matrices. (A) Vero cells (12 500 cells/well) were treated immediately after seeding into E-plates with 1∶14-diluted food extracts from milk (dotted grey line), carrot juice (dotted black line), baby food (grey line) or medium (black line). Characteristic growth phases of the cells were dynamically monitored every 15 min for 43 h. (B) Vero cells were exposed to ricin spiked into milk, carrot juice, baby food or medium, respectively. The indicated toxin concentrations are post-dilution concentrations. The viability of the cells is depicted as percentage of viable cells plotted against toxin concentrations in the different food matrices, measured after 24 h. (C) Vero cells were treated as described in (B). The viability of the cells is depicted as percentage of viable cells plotted against toxin concentrations in the different food matrices, measured after 42 h. (D) Vero cells were incubated with different dilutions of *Ricinus communis*-containing fertilizer extract (1∶3.5, black; 1∶14 dark grey; 1∶56 light grey; 1∶224, hatched) either without treatment (native), preincubated with 6 µg polyclonal anti-ricin IgY for 1.5 h at 37°C (+Ab) or heated for 30 min at 95°C (heated). For guidance, Vero cells were treated in parallel with different concentrations of purified ricin (white bars, 230 ng/mL, 2.3 ng/mL, 0.23 ng/mL). The viability of the cells after 21 h is depicted as percentage of the viability of the untreated control cells (100%). Data shown are exemplary data out of two independent experiments showing similar results.

In order to detect ricin in the presence of complex food matrices, we performed a series of experiments to find out how much food matrix would be tolerated by the cells (data not shown). In the final protocol the food matrices were diluted 1∶14 in medium and a clarified homogenate was added to the cells. As shown in Figure 5A, cells treated with 1∶14-diluted carrot juice and milk showed a similar proliferation pattern as untreated cells. Diluted baby food extract, however, interfered more strongly with cell proliferation leading to a decelerated growth. To determine the ability of the real-time cytotoxicity assay to detect ricin from complex food matrices, serial dilutions of ricin were spiked into homogenized food extracts (from milk, carrot juice and baby food) and incubated with the cells for 24 h (Figure 5B) and 42 h (Figure 5C). Functionally active toxin was detectable from all complex matrices spiked with ricin after 24 h and 42 h of incubation. The sigmoidal dose-dependent response curve for ricin spiked into diluted carrot juice was very similar to the medium control, while the response curve for ricin spiked into diluted milk significantly shifted to higher concentrations (Figure 5B and 5C). Based on these data, the IC50 for ricin spiked into diluted carrot juice was 0.4 ng/mL (corresponding to 5.6 ng/mL in the undiluted matrix) and for diluted milk 14.3 ng/mL (corresponding to 200 ng/mL in the undiluted matrix, respectively; Table S4). For ricin spiked into the difficult matrix baby food first results could be visualized as drop in CI within 24 h yielding an IC50 of about 0.4 ng/mL. However, the spreading of the dose-dependent response curve was marginal. In this case incubation for 42 h resulted in a reasonable dose-dependent response curve and delivered an IC50 of 0.1 ng/mL for the diluted matrix (corresponding to 1.4 ng/mL in the undiluted matrix; Table S4). As shown in Table S4, for all ricin-spiked food matrices the IC50 obtained after 24 h is about 3–4 times higher than the IC50 obtained after 42 h.

In order to show that the real-time cytotoxicity assay can be used to screen for active ricin in environmental samples, we tested different lots of organic fertilizer containing *Ricinus communis* material (castor meal). During the industrial production of castor oil, ricin is obtained as a by-product in the castor bean meal and has to be thoroughly heat-inactivated. However, in the past there have been reports of dog poisoning, presumably caused by the accidental ingestion of fertilizer that was insufficiently heat-treated [2], [35]. Having been involved in a similar case of dog poisoning in Germany, we tested extracts of the suspected *Ricinus communis*-containing fertilizer using the real-time cytotoxicity assay. An example of one fertilizer, Figure 5D, shows that different dilutions of the fertilizer extract induced cytotoxicity in Vero cells. Using a 1∶14 dilution of the buffered fertilizer extract, about 37% of the Vero cells were alive after 21 h. Preincubation of this dilution with anti-ricin IgY (+Ab) prevented cell death. Similarly, heat treatment of the extract for 30 min at 95°C also prevented cytotoxicity, both results indicate that the toxic effect on the cells is caused by functionally active ricin contained in the fertilizer extract (for guidance, different concentrations of purified ricin were tested in parallel).

## Discussion

We report a sensitive and rapid real-time cytotoxicity assay for the detection and quantification of functionally active ricin and agglutinin based on impedance sensor technology. This specific method was applicable for the detection of *Ricinus communis* material from complex matrices, e.g. food and organic fertilizer.

Classical functional assays for ricin include animal bioassays, endpoint cytotoxicity assays, adenine release assays, cell-free translation assays or enzyme-linked lectin assays (for an overview, see Table S1). For AB toxins like ricin, it has been shown previously that neither the isolated A-chain nor the isolated B-chain are toxic alone [36], [37]. Furthermore, there are hints that under certain denaturating conditions the sugar-binding property of the B-chain is destroyed, whereas the enzymatic activity of the A-chain is retained, even in the presence of an intact disulfide bond between the A- and B-chain [38]. This indicates the necessity for functional assays that show the presence AND the functional activity of both subunits. According to this criterion, several of the assays known are suitable to detect active ricin, e.g. animal bioassays and cell-based cytotoxicity assays. However, these assays have certain limitations, because animals are required [26], [28], [39], because of long cell cultivation and assay times [33], the need for transfection to generate fluorescently labeled reporter cells [32], or the use of radioactive materials [31]. The ricin detection based on the RT-CES system works label-free, and does not require radioactivity, enzymatic assays or transfection steps. Depending on the toxin concentration and the matrix used, active ricin can be detected between 3.5 to 60 h, which is comparable to previously described assays and faster than most endpoint cytotoxicity assays. High sensitivity, however, is reached after 24 h, with an IC50 of 0.4 ng/mL which is in the range of endpoint assays (Table S1). Compared to endpoint assays, the most important advantage of the impedance measurement is the online monitoring of cellular status, which offers the possibility to standardize the assay. Impedance read-outs in the RT-CES system depend on cell number, cell size and morphology, as well as adhesion characteristics of the cell line chosen. Ricin action was recorded online after cell attachment and lag-phase, and the kinetic control of the cellular status before and after ricin action revealed continuous information about growth, morphological changes and cell death. Therefore, abnormal first growth phases were an indicator of problems in assay performance. Even slight changes in cell culture conditions could be visualized and were used to optimize the assay. The CI values for the attachment and lag-phase were used as quality parameters to reduce assay variability. Based on this, the intra-assay variability was routinely below 13% for ricin tested at 1 ng/mL or below and the inter-assay variability was between 6 and 30% (24 h incubation, Table S2).

In recent years, highly sophisticated mass-spectrometry-based methods for detection and quantification of ricin have been introduced [29], [40], [41]. These methods combine an immunoaffinity enrichment of ricin, e.g. via its B-chain, followed by detection and/or quantification of adenine release by the A-chain. Strictly speaking, these assays detect the presence of the B-chain (not its activity), plus the activity of the A-chain. The advantage of these assays is their high precision – they are able to discriminate and quantify the highly homologous ricin and agglutinin down to a few fmol/mL, even in food matrices [40]. This data cannot be obtained by the cellular assays described or by conventional immunological assays. Since these technologies require high-end mass spectrometric equipment and specialised technical expertise, they might not be useful for broad application in routine laboratories. The impedance technology described, however, is easy to use and reduces cell culture work-load to a minimum, combined with low operative cost and effort. Therefore, this technology might complement standard routine ricin detection approaches.

The impedance technology might also be useful for screening of functionally blocking anti-ricin monoclonal antibodies or small molecule inhibitors, since multiple samples can be analyzed in parallel in a 96-well or 384-well format, thereby further improving endpoint cytotoxicity screening approaches [42], [43]. Combined with automated liquid handling platforms, high-throughput screening and objective quantitative data analysis is possible within a minimal amount of time.

On a qualitative basis, the impedance technology allows the comparison of the toxicity of substances, as shown here for ricin and agglutinin. At the time points of highest sensitivity (24–48 h), ricin was 60- to 75-fold more toxic than the closely related agglutinin, and the data are in accordance with previous data in the literature [12], [13]. For related plant AB toxins, abrin and abrin agglutinin, it was shown that the reduced toxicity of abrin agglutinin is associated with amino acid substitutions in a conserved region of ribosome inactivating proteins, resulting in a modified three-dimensional structure, which prevents an affine substrate binding [44]. With respect to ricin and agglutinin, the difference in toxicity is not understood on a molecular basis.

Since the ricin-producing plant *R. communis* is used on an industrial scale for the production of castor oil, there is concern that as a by-product, ricin could be used to deliberately contaminate the food supply chain. Furthermore, the castor meal itself is used as a cheap additive in organic fertilizers, since it is a rich source of nitrate. Therefore, it was important to show the applicability of the impedance technology on food matrices and fertilizer samples. Ricin was detected in concentrations between 5.6 ng/mL in carrot juice or baby food and 200 ng/mL in milk. It is known that the functional activity of ricin is inhibited by various sugars, e.g. lactose and galactose [45]. The predominant carbohydrate in milk is lactose, which interacts with the ricin B-subunit and therefore interferes with ricin detection in functional assays. Generally, the real-time cytotoxicity assay described here showed only minor interference with matrix compounds from the food tested. The assay was also applicable for the detection of ricin from fertilizer samples and actually turned out to be useful for forensic analysis. Having been involved in a case of dog poisoning in Germany in 2010, we were able to show that the deceased animal had taken up fertilizer containing significant amounts of active ricin which was obviously not correctly heat-inactivated during the production process [46]. Similar cases have been documented worldwide [2], [35], [47].

In conclusion, the impedance sensor technology presented offers a fast and label-free real-time monitoring of the functional activity of the ricin holo-toxin, using cell death as read-out as a consequence of both the sugar-binding and the enzymatic activity of the molecule. Combined with ELISA and precise mass-spectrometry assays, it will be useful to complement the information obtained from analyzing real sample materials. The method is easy to use in routine laboratories, requires minimum hands-on time and can be automated for high-throughput screening for anti-ricin inhibitory substances.

## Materials and Methods

### Material

Ricin (purity 98%) was purified along with agglutinin (purity 96%) either from seeds of *Ricinus communis carmencita* or from an unknown cultivar according to standard procedures [48]. Abrin was purchased from Toxin Technology (Sarasota, FL, USA). *Dolichos biflorus* agglutinin (DBA) and *Triticum vulgaris* agglutinin (TVA) were obtained from Sigma-Aldrich (Munich, Germany). Milk (UHT-milk, 3.5% fat, pH 7, brand: Tip, Metro, Düsseldorf, Germany), carrot juice (with lemon juice and ascorbic acid, pH 5, brand: Drink, Kaiser's Tengelmann, Viersen, Germany) and baby food (blueberry and apple dessert, pH 3, brand: Hipp, Pfaffenhofen, Germany) were purchased from a local retail store. Anti-ricin-specific polyclonal chicken IgY has been described elsewhere [49].

### Cell culture conditions

African green monkey (Vero) cells were acquired from the American Type Culture Collection (ATCC, Manassas, VA, USA). Cells grown to 50–80% confluent monolayers (∼25 000 cells/cm^2^), at passages 153 to 163, were used for the experiments. Before each assay, Vero cells were trypsinized with Trypsin/EDTA solution (0.2%/0.02% (w/v)) in PBS, centrifuged and resuspended in the appropriate medium volume and counted using a hemacytometer. Cells were cultured in Dulbecco's Modified Eagle Medium (DMEM, Biochrom, Berlin, Germany) containing 10% fetal bovine serum (Invitrogen, Karlsruhe, Germany), L-glutamine (2 mM, Biochrom, Berlin, Germany), 100 IU/mL penicillin and 100 µg/mL streptomycin (PAA Laboratories, Pasching, Austria). Cells were incubated at 37°C in a humidified 5% CO_2_ atmosphere.

### Cell proliferation test

For real-time monitoring of Vero cell proliferation, the baseline impedance of each sensor well in a 96-well E-plate (Roche Diagnostics, Mannheim, Germany) was measured by addition of 105 µl cell culture medium. Vero cells were adjusted to the appropriate concentrations (serial dilutions from 666 666 to 5 208 cells/mL) in cell culture medium, and the baseline medium in the E-plate well was substituted by 105 µl of cell suspension. Empty wells were filled with 105 µl PBS to avoid desiccation. Edge wells were not used in order to reduce variability resulting from edge effects [50]. The CI was automatically determined every 15 min by the RT-CES system (Roche Diagnostics, Mannheim, Germany), over a period up to 90 h. CI as a quantitative measure of the status of the cells in an electrode-containing well is calculated by the software according to [34]:
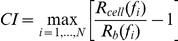
Where *R_cell_* stands for resistance of the electrode with attached cells, *R_b_* stands for resistance of the electrode without attached cells and *N* is the number of the frequency points at which the impedance is measured.

### RT-CES cytotoxicity assay

After baseline measurement of the E-plate, a Vero cell suspension containing 12 500 cells/well in a volume of 75 µl was seeded into the E-plates. Immediately after the seeding of cells into the wells (i.e. without prior attachment of the cells onto the plate), ricin (either native or denatured), agglutinin, complex matrices, extracts of *R. communis*-containing fertilizer or other plant lectins at given concentrations were added onto the cells in a volume of 30 µl. Each sample was measured at least in duplicate. The CI was automatically determined every 15 min by the RT-CES system (Roche Diagnostics, Mannheim, Germany), over a period of up to 24–42 h. During the incubation, only live cells attached onto the plate and showed a vigorous proliferation (equivalent to an increase in impedance and CI). Depending on the toxin concentration present on the cells, the proliferation was terminated after different time points, followed by detachment and cell death (equivalent to a drop of impedance and CI). To specifically block ricin and agglutinin cytotoxicity, the test compounds were preincubated with 880 µg anti-ricin IgY for 1.5 h at 37°C on a shaker.

### MTT cytotoxicity assay

To determine ricin and agglutinin cytotoxicity in a colorimetric endpoint assay, an MTT (3-[4,5-Dimethylthiazol-2-yl]-2,5-diphenyltetrazolium bromide) assay was performed as described previously [14], [51]. Briefly, Vero cells (10 000 cells/well) were cultured in a 96-well plate for 18 h. Cells were treated with toxin dilutions for 2 h, washed and further incubated for 20 h in medium. The endpoint viability of the Vero cells was quantified using the CellTiter96 Non-Radioactive Cell Proliferation Assay (Promega, Madison, WI, USA).

### Heat inactivation of ricin and analysis of *R. communis*-containing fertilizer

Ricin was diluted in PBS to a concentration of 20 000 ng/ml. In order to guarantee optimal heat transfer, 100 µl solution were incubated in a thin-wall PCR tube for 30 min at 95°C in a PCR cycler, cooled on ice and then used for the experiments, as described for our recent experiments on the stability of botulinum neurotoxins [52].

*Ricinus communis*-containing fertilizer was ground with a coffee grinder. 2 g of ground fertilizer were mixed with 20 ml of PBS and rotated for 2 h at room temperature followed by centrifugation. The supernatant was filtered through a 70 µm sieve and stored at 4°C. For heat inactivation of the *R. communis*-containing fertilizer, 100 µl of the extract was heated for 30 min at 95°C, as indicated above.

### Analysis of complex food samples

Serial dilutions of ricin were spiked into milk, carrot juice or baby food. Spiked and unspiked food samples were diluted 1∶4 (v/v) in cell culture medium, centrifuged and filtered through a 0.45 µm filter.

### Data analysis

All calculations and figures were obtained using GraphPad Prism software 5.01 (GraphPad, San Diego, CA, USA). The curve fitting of the standard curves was a nonlinear regression: log(inhibitor) vs. response-variable slope (four parameters). Cell viability was either depicted as CI value of the RT-CES system over time, or converted into percent (%) of the control cells alive over toxin concentration. To this end, the CI value of nontreated cells at several time points was set to 100%, and for a given time point the ratio of CI values of toxin-treated cells to nontreated cells was calculated. *In vitro* cytotoxicity at 50% (IC50) was defined as the toxin concentrations required to reduce cell viability by 50% compared to untreated control cells at various time points. Within- and between-run precision was evaluated by the measurement of cytotoxicity of serial dilutions of ricin after 24 h or 42 h, respectively: for within-run precision serial dilutions of ricin (1 ng/mL to 0.03 ng/mL) were performed in four replicates and measured on one day; for between-run precision serial dilutions of ricin were performed on four different days and the results were used for calculation. The coefficient of variation (CV) equals the standard deviation of the concentration-dependent CI values divided by the mean of the CI values.

## Supporting Information

Figure S1 **Dynamic monitoring of Vero cell proliferation depending on different cell culture conditions.** (A) To illustrate the different growth characteristics of Vero cells depending on culture conditions prior to the cytotoxicity assay, the cells were grown in different densities in culture flasks at 10 000 cells/cm^2^ (dashed line), 25 000 cells/cm^2^ (black line) and 100 000 cells/cm^2^ (dotted line). Then Vero cells were trypsinized and seeded in a 96-well E-plate at 12 500 cells/well. Cell proliferation was dynamically monitored every 15 min for 22 h. (B) Vero cells were grown at a density of 25 000 cells/cm^2^ in culture flasks before the cytotoxicity assay and removed by trypsinization with either Trypsin (0.2%) containing EDTA (0.02%, black line) or EDTA (0.07%, dotted line). Then the cells were seeded in a 96-well E-plate at 12 500 cells/well and proliferation was dynamically monitored every 15 min for 22 h.
(TIF)

Table S1 **Comparison of real-time ricin cytotoxicity assay with other functional ricin detection methods.** The table depicts information on different functional assays for ricin detection highlightening their detection principle, measurement parameters, assay time, specificity and the application to detect ricin from complex matrices.
(PDF)

Table S2 **Within-run and between-run precision for ricin detection using the novel real-time cytotoxicity assay.** The precision of the optimized real-time cytotoxicity assay was evaluated by the determination of the coefficient of variation (CV) analyzing the cytotoxicity data obtained by measuring serial dilutions of ricin on Vero cells after 24 h or 42 h, respectively: For within-run precision serial dilutions of ricin (1 ng/mL to 0.03 ng/mL) were performed in four replicates and measured on one day; for between-run precision serial dilutions of ricin were performed on four different days. The CV near the IC50 value is highlighted in grey.
(PDF)

Table S3 **Within-run and between-run precision for ricin detection using the conventional endpoint cytotoxicity assay (MTT assay).** The precision of the MTT assay was evaluated by the determination of the coefficient of variation (CV) analyzing the cytotoxicity data obtained by measuring serial dilutions of ricin on Vero cells after 45 h: For within-run precision serial dilutions of ricin (100 ng/mL to 0.4 ng/mL) were performed in four replicates and measured on one day; for between-run precision serial dilutions of ricin were performed on four different days. The CV near the IC50 value is highlighted in grey.
(PDF)

Table S4 **IC50 values for ricin spiked into different food matrices.** Vero cells were exposed to ricin spiked into milk, carrot juice, baby food or medium, respectively. The IC50 values of serial dilutions of ricin in the complex matrices (see Figure 5) after 24 h and 42 h are shown.
(PDF)
