# Supplementary figures and images for: Real-Time Cytotoxicity Assay for Rapid and Sensitive Detection of Ricin from Complex Matrices
Source: PLoS One. 2012 Apr 19;7(4):e35360. doi: 10.1371/journal.pone.0035360 (PMC3330811; doi:10.1371/journal.pone.0035360)

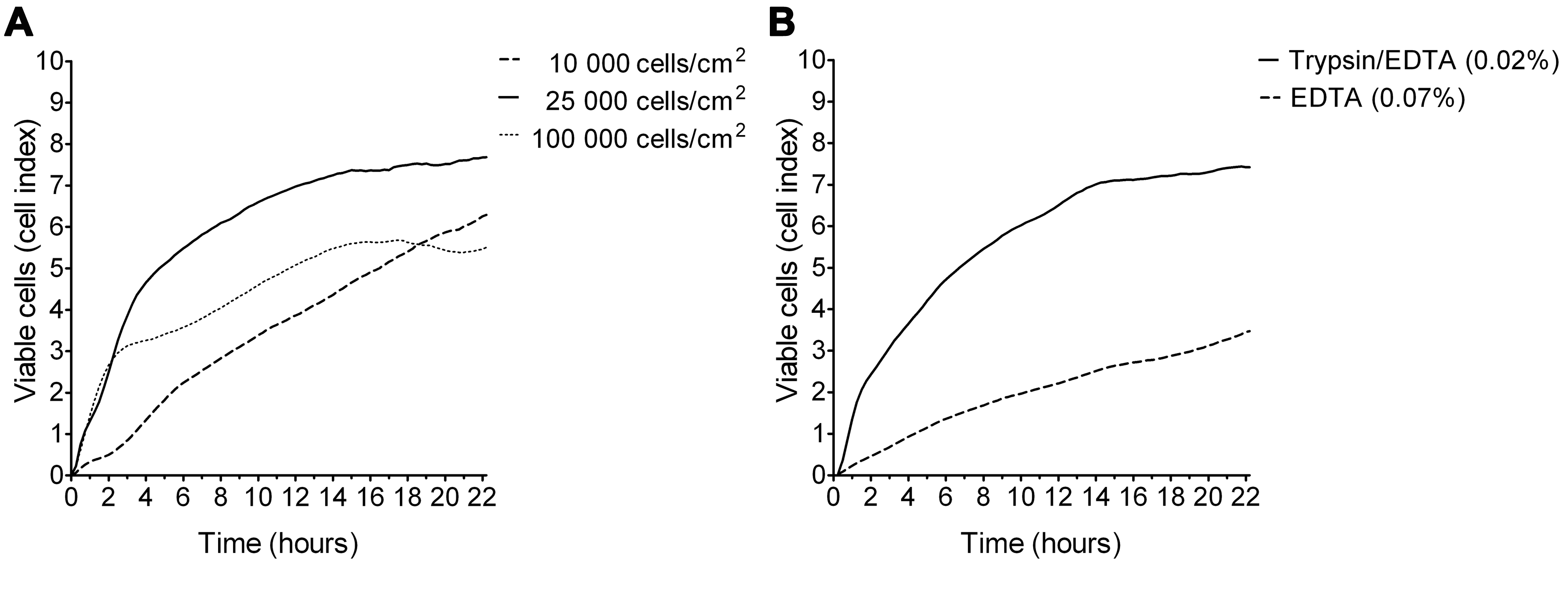

Supplement: Figure S1 — Dynamic monitoring of Vero cell proliferation depending on different cell culture conditions. (A) To illustrate the different growth characteristics of Vero cells depending on culture conditions prior to the cytotoxicity assay, the cells were grown in different densities in culture flasks at 10 000 cells/cm2 (dashed line), 25 000 cells/cm2 (black line) and 100 000 cells/cm2 (dotted line). Then Vero cells were trypsinized and seeded in a 96-well E-plate at 12 500 cells/well. Cell proliferation was dynamically monitored every 15 min for 22 h. (B) Vero cells were grown at a density of 25 000 cells/cm2 in culture flasks before the cytotoxicity assay and removed by trypsinization with either Trypsin (0.2%) containing EDTA (0.02%, black line) or EDTA (0.07%, dotted line). Then the cells were seeded in a 96-well E-plate at 12 500 cells/well and proliferation was dynamically monitored every 15 min for 22 h. (TIF) [file pone.0035360.s001.tif]
