# Supplementary material for: Real-Time Cytotoxicity Assay for Rapid and Sensitive Detection of Ricin from Complex Matrices
Source: PLoS One. 2012 Apr 19;7(4):e35360. doi: 10.1371/journal.pone.0035360 (PMC3330811; doi:10.1371/journal.pone.0035360)
Supplement: Table S1 — Comparison of real-time ricin cytotoxicity assay with other functional ricin detection methods. The table depicts information on different functional assays for ricin detection highlightening their detection principle, measurement parameters, assay time, specificity and the application to detect ricin from complex matrices. (PDF) [file pone.0035360.s002.pdf]

Table S-1. Comparison of real-time ricin cytotoxicity assay with other functional ricin detection methods.

The table depicts information on different functional assays for ricin detection highlightening their detection principle, measurement parameters, assay time, specificity and the application to detect ricin from complex matrices.

Assay	Target	Detection principle	Ricin detection	Reference value	Assay time	Specificity	Complex matrices	Ref.
Cytotoxicity assay (real-time)	Ricin A Ricin B	real-time monitoring of cell death: impedance measurement	0.4 ng/mL	IC50	24 h	specific ricin antibody blockade	milk, carrot juice, baby food	this paper
			0.1 ng/mL	IC50	42 h			
Cytotoxicity assay (endpoint)	Ricin A Ricin B	endpoint determination of cell death: determination of lactate dehydrogenase concentration	0.01 ng/mL	blank plus 3×standard deviation	16–17 h	specific ricin antibody blockade	orange juice, milk, coffee, soda, infant formula	[1]
	Ricin A Ricin B	endpoint determination of cell death: incorporation of ^{14}C-Leucin into proteins	0.8 ng/mL	IC50	4 h	specific ricin antibody blockade		[2]
	Ricin A Ricin B	endpoint determination of cell death: expressing green fluorescent protein	0.23 ng/mL	IC50	18–22 h			[3]
			1.8 ng/mL	IC50	6 h			
	Ricin A Ricin B	endpoint determination of cell death: cell lysis (MTT-Assay) and others	0.1 ng/mL	IC50	28 h			[4]
Mouse assay	Ricin A Ricin B	mouse body temperature, lack of response to tactile stimuli	5 000 ng	one concentra-tion tested	14–16 h	specific ricin antibody blockade		[5]
	Ricin A Ricin B	lethality of mouse	60 ng	LD50	10–11 h		spring water, apple juice, milk	[6,7]
Adenine release assay	Ricin A Ricin B	immunoaffinity enrichment of ricin using an anti-ricin B antibody combined with mass spectrometry detection of adenine release from nucleic acid substrate by ricin A	0.1–0.2 ng/mL	Signal to noise ratio >3–10	26 h	tryptic digestion of captured antigen followed by mass spectrometry analysis	milk, tap or bottled water, apple juice, orange juice, serum, saliva	[8,9]
			0.3–0.6 ng/mL	Signal to noise ratio >3-10	6 h			
		immunoaffinity enrichment of ricin using an anti-ricin polyclonal antibody combined with mass spectrometry detection and quantification of adenine release from nucleic acid substrate by ricin A	64 ng/ml	Lower limit of detection	~ 5 h	tryptic digestion of captured antigen followed by sequencing and absolute quantification of ricin / agglutinin	milk, tap water, apple juice, orange juice	[10]

	Ricin A	cleavage of adenine from nucleic acid substrate measured by HPLC and mass spectrometry	5.8 ng/mL	one concentration tested	1 h			[11,12]
	Ricin A	cleavage of adenine from nucleic acid substrate measured by Ruthenium coupled probe	0.1 ng/mL	blank plus 3×standard deviation	3–4 h	specific ricin activity enhancement by antibodies		[13]
	Ricin A	cleavage of adenine from nucleic acid substrate measured by molecular beacon	14 ng/mL	Lower limit of detection	~ 10 h			[14]
Cell-free translation assay	Ricin A	luciferase activity as reporter for protein translation	7.2 ng/mL	IC50	0.5 h	specific ricin antibody blockade; positively tested: saponin, abrin, viscumin	ground beef, low fat milk, liquid chicken egg	[15,16,17]
Enzyme linked lectin assay	Ricin B	enzyme-linked lectin-assay, binding to asialofetuin	4 ng/mL	Signal to noise ratio >3	16 h			[18] and unpublished data (D. Pauly)

References Supporting Information

1. Brzezinski JL, Craft DL (2007) Evaluation of an in vitro bioassay for the detection of purified ricin and castor bean in beverages and liquid food matrices. J Food Prot 70: 2377-2382.
2. Colombatti M, Johnson VG, Skopicki HA, Fendley B, Lewis MS, et al. (1987) Identification and characterization of a monoclonal antibody recognizing a galactose-binding domain of the toxin ricin. J Immunol 138: 3339-3344.
3. Halter M, Almeida JL, Tona A, Cole KD, Plant AL, et al. (2009) A mechanistically relevant cytotoxicity assay based on the detection of cellular GFP. Assay Drug Dev Technol 7: 356-365.
4. Oda T, Komatsu N, Muramatsu T (1997) Cell lysis induced by ricin D and ricin E in various cell lines. Biosci Biotechnol Biochem 61: 291-297.
5. Beyer NH, Kogutowska E, Hansen JJ, Engelhart Illigen KE, Heegaard NH (2009) A mouse model for ricin poisoning and for evaluating protective effects of antiricin antibodies. Clin Toxicol (Phila) 47: 219-225.
6. Fodstad Ø, Olsnes S, Pihl A (1976) Toxicity, distribution and elimination of the cancerostatic lectins abrin and ricin after parenteral injection into mice. Br J Cancer 34: 418-425.
7. Garber EA (2008) Toxicity and detection of ricin and abrin in beverages. J Food Prot 71: 1875-1883.
8. Becher F, Duriez E, Volland H, Tabet JC, Ezan E (2007) Detection of functional ricin by immunoaffinity and liquid chromatography-tandem mass spectrometry. Anal Chem 79: 659-665.
9. Kalb SR, Barr JR (2009) Mass spectrometric detection of ricin and its activity in food and clinical samples. Anal Chem 81: 2037-2042.
10. McGrath SC, Schieltz DM, McWilliams LG, Pirkle JL, Barr JR (2011) Detection and quantification of ricin in beverages using isotope dilution tandem mass spectrometry. Anal Chem 83: 2897-2905.
11. Bevilacqua VL, Nilles JM, Rice JS, Connell TR, Schenning AM, et al. (2010) Ricin activity assay by direct analysis in real time mass spectrometry detection of adenine release. Anal Chem 82: 798-800.
12. Hines HB, Brueggemann EE, Hale ML (2004) High-performance liquid chromatography-mass selective detection assay for adenine released from a synthetic RNA substrate by ricin A chain. Anal Biochem 330: 119-122.
13. Keener WK, Rivera VR, Young CC, Poli MA (2006) An activity-dependent assay for ricin and related RNA N-glycosidases based on electrochemiluminescence. Anal Biochem 357: 200-207.
14. Roday S, Sturm MB, Blakaj D, Schramm VL (2008) Detection of an abasic site in RNA with stem-loop DNA beacons: application to an activity assay for Ricin Toxin A-Chain. J Biochem Biophys Methods 70: 945-953.
15. He X, Lu S, Cheng LW, Rasooly R, Carter JM (2008) Effect of food matrices on the biological activity of ricin. J Food Prot 71: 2053-2058.
16. Langer M, Rothe M, Eck J, Mockel B, Zinke H (1996) A nonradioactive assay for ribosome-inactivating proteins. Anal Biochem 243: 150-153.
17. Hale ML (2001) Microtiter-based assay for evaluating the biological activity of ribosome-inactivating proteins. Pharmacol Toxicol 88: 255-260.
18. Vang O, Larsen KP, Bøg-Hansen TC (1986) A new quantitative and highly specific assay for lectin binding activity. In: Bøg-Hansen TC, van Driessche E, editors. Lectins: Biology, Biochemistry, Clinical Biochemistry. Berlin: de Gruyter. pp. 638-644.
