# Supplementary material for: Real-Time Cytotoxicity Assay for Rapid and Sensitive Detection of Ricin from Complex Matrices
Source: PLoS One. 2012 Apr 19;7(4):e35360. doi: 10.1371/journal.pone.0035360 (PMC3330811; doi:10.1371/journal.pone.0035360)
Supplement: Table S2 — Within-run and between-run precision for ricin detection using the novel real-time cytotoxicity assay. The precision of the optimized real-time cytotoxicity assay was evaluated by the determination of the coefficient of variation (CV) analyzing the cytotoxicity data obtained by measuring serial dilutions of ricin on Vero cells after 24 h or 42 h, respectively: For within-run precision serial dilutions of ricin (1 ng/mL to 0.03 ng/mL) were performed in four replicates and measured on one day; for between-run precision serial dilutions of ricin were performed on four different days. The CV near the IC50 value is highlighted in grey. (PDF) [file pone.0035360.s003.pdf]

Table S-2. Within-run and between-run precision for ricin detection using the novel real-time cytotoxicity assay

The precision of the optimized real-time cytotoxicity assay was evaluated by the determination of the coefficient of variation (CV) analyzing the cytotoxicity data obtained by measuring serial dilutions of ricin on Vero cells after 24 h or 42 h, respectively: For within-run precision serial dilutions of ricin (1 ng/mL to 0.03 ng/mL) were performed in four replicates and measured on one day; for between-run precision serial dilutions of ricin were performed on four different days. The CV near the IC50 value is highlighted in grey.

**Ricin ng/mL**	**24 hours**		**42 hours**	
	**Within-run CV**	**Between-run CV**	**Within-run CV**	**Between-run CV**
1	13%	23%	30%	40%
0.5	4%	21%	6%	59%
0.25	9%	30%	9%	49%
0.125	6%	26%	11%	28%
0.06	2%	6%	2%	31%
0.03	5%	10%	2%	7%
