# Supplementary material for: Real-Time Cytotoxicity Assay for Rapid and Sensitive Detection of Ricin from Complex Matrices
Source: PLoS One. 2012 Apr 19;7(4):e35360. doi: 10.1371/journal.pone.0035360 (PMC3330811; doi:10.1371/journal.pone.0035360)
Supplement: Table S3 — Within-run and between-run precision for ricin detection using the conventional endpoint cytotoxicity assay (MTT assay). The precision of the MTT assay was evaluated by the determination of the coefficient of variation (CV) analyzing the cytotoxicity data obtained by measuring serial dilutions of ricin on Vero cells after 45 h: For within-run precision serial dilutions of ricin (100 ng/mL to 0.4 ng/mL) were performed in four replicates and measured on one day; for between-run precision serial dilutions of ricin were performed on four different days. The CV near the IC50 value is highlighted in grey. (PDF) [file pone.0035360.s004.pdf]

Table S-3. Within-run and between-run precision for ricin detection using the conventional endpoint cytotoxicity assay (MTT assay)

The precision of the MTT assay was evaluated by the determination of the coefficient of variation (CV) analyzing the cytotoxicity data obtained by measuring serial dilutions of ricin on Vero cells after 45 h: For within-run precision serial dilutions of ricin (100 ng/mL to 0.4 ng/mL) were performed in four replicates and measured on one day; for between-run precision serial dilutions of ricin were performed on four different days. The CV near the IC50 value is highlighted in grey.

**Ricin ng/mL**	**45 hours**	
	**Within-run CV**	**Between-run CV**
100	13%	18%
33	15%	17%
11	11%	21%
3.7	28%	23%
1.2	16%	23%
0.4	11%	23%
