# Supplementary material for: Real-Time Cytotoxicity Assay for Rapid and Sensitive Detection of Ricin from Complex Matrices
Source: PLoS One. 2012 Apr 19;7(4):e35360. doi: 10.1371/journal.pone.0035360 (PMC3330811; doi:10.1371/journal.pone.0035360)
Supplement: Table S4 — IC50 values for ricin spiked into different food matrices. Vero cells were exposed to ricin spiked into milk, carrot juice, baby food or medium, respectively. The IC50 values of serial dilutions of ricin in the complex matrices (see Figure 5) after 24 h and 42 h are shown. (PDF) [file pone.0035360.s005.pdf]

Table S-4. IC50 values for ricin spiked into different food matrices.

Vero cells were exposed to ricin spiked into milk, carrot juice, baby food or medium, respectively. The IC50 values of serial dilutions of ricin in the complex matrices (see Figure 5) after 24 h and 42 h are shown.

**Matrix**	**IC50 after 24 hours**		**IC50 after 42 hours**	
	1:14 diluted matrix added to the cells	Undiluted matrix	1:14 diluted matrix added to the cells	Undiluted matrix
Medium	0.4 ng/mL	5.6 ng/mL	0.1 ng/mL	1.4 ng/mL
Milk	14.3 ng/mL	200.2 ng/mL	4.2 ng/mL	58.8 ng/mL
Carrot juice	0.4 ng/mL	5.6 ng/mL	0.1 ng/mL	1.4 ng/mL
Baby food	0.4 ng/mL	5.6 ng/mL	0.1 ng/mL	1.4 ng/mL
